# Effects of prenatal heat stress on birth weight and birth weight genetic parameters in German Holstein calves

**DOI:** 10.3168/jdsc.2023-0381

**Published:** 2023-08-19

**Authors:** T. Yin, K. Halli, S. König

**Affiliations:** Institute of Animal Breeding and Genetics, Justus-Liebig-University of Gießen, 35390 Gießen, Germany

## Abstract

The aim of this study was to infer the effects of heat stress (HS) during late gestation of dams on phenotypes and on direct and maternal genetic parameters for birth weight (BiW). We considered 171,221 Holstein calves kept in 56 large-scale co-operator herds. For a clear separation of maternal effects, only calves from dams with at least 3 offspring were included in the analyses. The genotype data set comprised 41,143 SNPs from 1,883 Holstein bulls. Temperature-humidity indices (THI) during the last 8 wk of gestation were calculated in each herd to reflect prenatal HS. A further prenatal HS descriptor was the first principal component (PC1) from principal component analysis considering the daily THI during the last 56 d of gestation. Regression coefficients of BiW on prenatal THI during the last 12 wk of gestation and PC1 were estimated in 13 consecutive phenotypic analyses. The strongest BiW decline was −0.63 kg per standardized THI, identified during 50 to 56 d before birth. A reaction norm model with weekly prenatal THI or PC1 nested within maternal genetic and maternal permanent environmental effects was defined to infer maternal sensitivity in response to prenatal THI alterations. Direct BiW heritabilities were close to 0.33 in the course of prenatal THI. Maternal BiW heritabilities marginally increased from 0.07 to 0.08 with increasing THI. Genetic correlations between maternal genetic effects at maximum HS levels and remaining THI were larger than 0.95, indicating the absence of genotype by time-lagged HS interactions. In contrast, maternal permanent environmental correlations between BiW at prenatal THI indicating HS with BiW at remaining THI substantially declined with increasing THI distances. Hence, from a herd management perspective, avoiding HS during the dry period of the dams will contribute to a slight increase in fetus growth.

## Highlights

• Prenatal heat stress slightly decreased calf birth weight.
• Direct and maternal heritabilities for calf birth weight were independent from prenatal heat stress effects.
• No genotype by climate interactions on calf birth weight were identified for maternal genetic effects.
• The maternal permanent environmental effects reflecting the dry cow management had slight influences on fetus growth.

## Graphical Abstract

**Figure:**
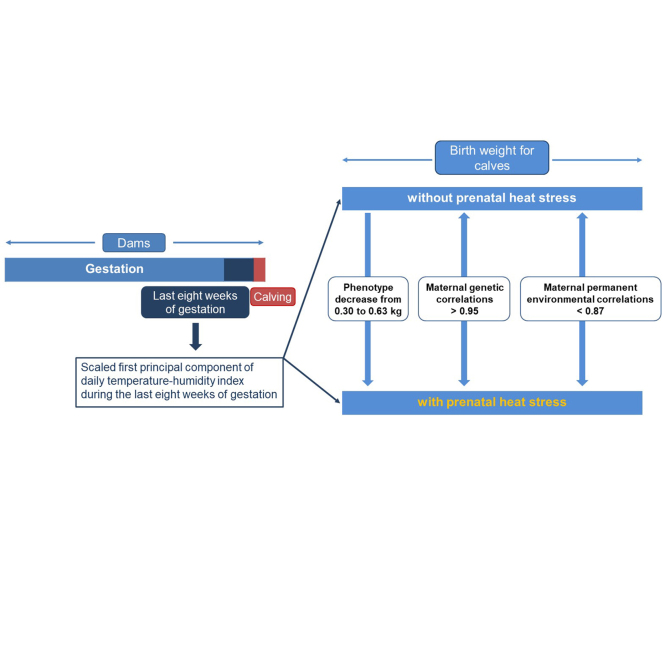
**Summary:** A data set including 171,221 Holstein calves was used to infer phenotypic effects and genetic parameters for calf birth weight (BiW) in response to prenatal heat stress (HS). Weekly temperature-humidity indices (THI) during the last 12 weeks of gestation and the first principal component (PC1) considering daily THI during the last 8 weeks were defined as prenatal HS descriptors. Phenotypically, the decline of BiW due to prenatal HS ranged from -0.30 to -0.63 kg per scaled weekly THI. Direct and maternal heritabilities for birth weight were constant along THI. Correlations between maternal genetic effects for BiW at the maximum HS level (PC1 = 1) with BiW at remaining THI were larger than 0.95, indicating the absence of genotype by time-lagged HS interactions. Maternal permanent environmental correlations substantially declined with increasing THI distances. Hence, avoiding HS during the dry period of the dams has a stronger impact on the fetuses' growth than genetic aspects, but also the statistically significant phenotypic BiW differences between the maternal HS and non-HS group were quite small from a practical perspective.

Birth weight (**BiW**) of calves is a complex breeding trait. From the dam perspective, smaller and lighter calves imply improved calving fitness and female fertility (e.g., reduced incidences for dystocia and female fertility disorders, and shorter gestation length; Johanson et al., 2011). Moderate to large genetic correlations among BiW, calving difficulties, and stillbirth in the range from 0.62 to 0.92 were pointed out by Eriksson et al. (2004). In contrast, from a calf perspective, Berger et al. (1992) observed the tendency that an extremely large or low BiW was associated with a decline in survival rates. Furthermore, BiW was genetically favorable correlated with body weights at later ages, including weaning weight, insemination weight, and weight at first calving (Coffey et al., 2006; Yin and König, 2018). In the ongoing time perspective, body weight at first insemination was genetically favorable correlated with the nonreturn rate of heifers (Yin and König, 2018) and female fertility after calving (Abdallah and McDaniel, 2000; Brotherstone et al., 2007).

Alternations of genetic (co)variance components for production traits along a temperature-humidity index (**THI**) gradient indicated genotype by climate interactions (Bohlouli et al., 2019). In addition to the immediate responses to heat stress (**HS**; i.e., HS effects directly at the test-day), effects of prenatal HS on production, fertility, and health in offspring were reported from an-across generation perspective (Halli et al., 2021; Kipp et al., 2021a; Yin et al., 2022). Genetic effects of prenatal HS were moderate for low heritability calf diseases. In this regard, the genetic correlations between same calf diseases at different maternal THI were in the range from 0.26 to 0.42 (Yin et al., 2022). The genotype by prenatal HS interaction variances for production traits were quite small (0 to 7% of the total variance) (Halli et al., 2021). Analogous to calf health traits, BiW is expressed at an early age stage directly at birth, implying a short period for prenatal HS effects from the across-generation perspective. With regard to the HS importance in the cow dry period, the last 2 mo of gestation contributed to 60% of the weight development of the fetus (Bauman and Currie, 1980). Phenotypically, BiW of calves from heat-stressed dams during the dry period were 2.6 to 13.0 kg lighter compared with calves without any prenatal HS (Wolfenson et al., 1988; do Amaral et al., 2009). However, the data sets used in these studies were quite small, based on specific experiments, and ignored the genetic components of prenatal HS.

The aim of this study was to estimate the effects of time-lagged HS on genetic components for BiW considering calves from large-scale commercial herds. The contents of this study included (1) the estimation of phenotypic regression coefficients of BiW on prenatal THI during the last 8 wk of gestation, and (2) the estimation of direct and maternal genetic parameters for BiW by alterations of weekly prenatal THI.

The calves were offspring from 46,328 dams kept in 56 large-scale co-operator herds located in the German federal states of Mecklenburg-West Pomerania and Berlin-Brandenburg. The calves born from October 2005 to June 2016 were weighed directly after birth using a scale. Phenotype data editing implied the exclusion of BiW records with >60 kg or <20 kg. The average BiW was 43.16 kg. For a clear estimation of maternal effects, we focused on repeated BiW measurements per dam, and only considered calf records from dams with more than 2 offspring from different parities. After data editing, the data set consisted of 171,221 calves, including 86,790 males and 84,431 females. The dams of the calves were from parities 1 to 13. On average, each dam had 3.70 progeny, in the range from 3 to 10 progeny per dam. The pedigree file included 264,235 animals and the ancestors of phenotyped calves were traced back to at least 3 generations.

The genotype data set included 1,931 bulls genotyped with the *Illumina BovineSNP50 v2 BeadChip* (397 bulls), or with the *Illumina Bovine Eurogenomics 10K low-density chip* (1,534 bulls). Bulls genotyped with the 10K chip were imputed to the 50K panel in official national genetic evaluations (Segelke et al., 2012). Genotyped bulls with genomic relationships larger than 0.95 and unrelated with the phenotyped calves were discarded. Finally, 41,143 SNPs were available for 1,883 genotyped bulls. The SNPs had a call rate larger than 0.95, a minor allele frequency larger than 0.05, and a nonsignificant deviation from Hardy-Weinberg equilibrium. Only SNPs located on autosomes were included in this study. Genotype quality control was performed using the preGSf90 program from the BLUPf90 package (Misztal et al., 2014). The SNP positions were coordinated according to the reference assembly ARS-UCD1.2.

We used the longitudes and altitudes of the herds and the weather stations to calculate pairwise distances (in km) between the herds and all public weather stations using the R-package geosphere (Hijmans et al., 2019). For each herd, the weather station in nearest distance was identified. We allocated 34 weather stations to 57 herds. The maximum distance between a herd and a weather station was 27.88 km, the minimum distance was 0.74 km, and the average distance was 14.17 km. Hourly temperature (T) and relative humidity (RH) data were used to compute hourly THI as follows (NRC, 1971):

THI=(1.8×T+32)−(0.55−0.0055×RH)×(1.8×T−26).

The prenatal weekly THI for the last 12 wk of gestation were calculated by averaging hourly THI. The analyzed prenatal weeks included 0 to 7 d (**WK1**), 8 to 14 d (**WK2**), 15 to 21 d (**WK3**), 22 to 28 d (**WK4**), 29 to 35 d (**WK5**), 36 to 42 d (**WK6**), 43 to 49 d (**WK7**), 50 to 56 d (**WK8**), 57 to 63 d (**WK9**), 64 to 70 d (**WK10**), 71 to 77 d (**WK11**), and 78 to 84 d (**WK12**) before birth. The average weekly THI across the 12 wk ranged from 10.38 to 74.28. As fetus growth is strongest during the last 2 mo of gestation (Bauman and Currie, 1980), principal component analysis was carried out for the daily THI during the last 56 d of gestation (0–56 d), with focus on the first principal component (**PC1**). The average THI for each week and PC1 were scaled as follows: $-1+2\left(\frac{THI-17}{72-17}\right),$ and $-1+2\left(\frac{PC1-\left(-15\right)}{11-\left(-15\right)}\right),$ respectively. The minimum and maximum THI for scaling weekly THI were set to 17 and 72, respectively. The scaled weekly THI (**sTHI**) and scaled PC1 (**sPC1**) were used in the ongoing analyses.

A linear mixed model was applied to study the effects of weekly prenatal sTHI and sPC1 on BiW. The regression coefficients of BiW on prenatal sTHI and sPC1 were estimated in 13 consecutive runs using the following statistical model 1:

[1] $$y=Xb+Ks+Mh+Qc_{hym}+e,$$

where **y** = vector of observations for BiW; **b** = vector of fixed effects including the overall mean, herd-birth-year (**HY**; 507 levels), sex and birth type (4 classes: pairwise combinations of males and females with singleton and twin births), gestation length classes of dams (class 1 = 260–269 d, class 2 = 270–274 d, class 3 = 275–279 d, class 4 = 280–284 d, class 5 = 285–289 d, and class 6 = 290–300 d), parity classes of dams (7 classes = parity 1 to 6, and >6), linear regression on weekly prenatal sTHI or sPC1; **s** = vector of random calf sire effects (2,510 sires); **h** = vector of random dam effects (46,328 dams); **c_hym_** = vector of random herd-birth-year-month effects (**HYM**; 5,290 levels); **e** = vector of random residual effects; and **X**, **K**, **M**, and **Q** = design matrices for **b**, **s**, **h**, and **c_hym_**, respectively.

A reaction norm model with weekly prenatal sTHI or sPC1 nested within maternal genetic and maternal permanent environmental effects was applied to estimate (co)variance components for BiW using the AIREML algorithm as implemented in the BLUPf90 program (Misztal et al., 2014). In matrix notation, the statistical model 2 was

[2] $$y=Xb+Zd+Wm+Sp_{m}+Qc_{hym}+e,$$

where **d** = vector of direct additive genetic effects; **m** = vector of maternal genetic effects for random intercepts (maternal intercepts) and slopes using scaled weekly sTHI or sPC1; **p_m_** = vector of maternal permanent environmental effects for random intercepts and slopes; and **Z**, **W**, and **S** = design matrices for **d, m**, and **p_m_**, respectively. The remaining terms in the model were the same as in model 1. The genetic relationship matrix was the **H** matrix, considering simultaneously the pedigree relationship matrix **A** and the weighted genomic relationship matrix (**Gw**; VanRaden, 2008; Legarra et al., 2009).

The regression coefficients for prenatal sTHI were in a range of −0.30 in WK1 to −0.63 in WK8, indicating unfavorable prenatal HS effects on BiW. For WK9 to WK12, the detrimental HS effects slightly declined (Table 1). Explicitly, a regression coefficient of −0.30 in WK1 implies a decrease of 0.30 kg in BiW per unit of sTHI increase. Hence, the predicted BiW for a calf with prenatal THI 72 in WK1 is 0.30 kg lighter than for a calf with prenatal THI 44.5. The slope for sPC1 was similar with the slope identified in WK4, because the sPC1 considered most of the THI variations during the last 56 d of gestation. The decline in BiW due to prenatal HS was intensively discussed in the review by Tao and Dahl (2013). Physiological explanations addressed shortened gestation length, reduced energy intake, and insufficient placental developments in dams with HS during the dry period.

**Table 1:** Regression coefficients of birth weight per changing unit of the weekly prenatal temperature-humidity index and first principal components of daily prenatal temperature-humidity index during late gestation

Covariate^1^	Regression coefficient^2^
sWK1	−0.30
sWK2	−0.37
sWK3	−0.48
sWK4	−0.53
sWK5	−0.55
sWK6	−0.56
sWK7	−0.61
sWK8	−0.63
sWK9	−0.61
sWK10	−0.59
sWK11	−0.54
sWK12	−0.51
sPC1	−0.52

1 sWK1 to sWK12 = scaled weekly prenatal temperature-humidity index during last 12 wk of gestation, respectively; sPC1 = scaled first principal components of daily prenatal temperature-humidity index during the last 0 to 56 d of gestation.

2 The standard error for all coefficients was 0.03; the *P*-value for all coefficients was <0.001.

The detrimental effects of prenatal HS on BiW increased gradually from WK1 to WK8, and decreased slightly from WK9 to WK12. The strongest detrimental effect was found in WK8. In contrast, with regard to impaired female fertility and productivity in lactating cows, and health in newborn calves, Kipp et al. (2021b) and Yin et al. (2022) identified HS in WK1 as the most important period. In dairy cows, the fetus growth rate is strongest during the last 2 mo of gestations (Bauman and Currie, 1980). In the present study, the detrimental effect of prenatal HS on BiW with a maximal difference of 0.63 kg between the calves exposed to prenatal HS and the control group from the thermoneutral zone (THI ~44.5) was smaller compared with differences ranging from 2.6 to 13.0 kg in experimental studies (Wolfenson et al., 1988; do Amaral et al., 2009). The larger BiW differences in the experimental studies might be due to the pronounced HS effects as prevalent in tropical and subtropical climates, but Germany is located in a temperate climatic zone. For example, Adin et al. (2009) calculated an average daily THI of 77.9 during the experimental period (about 5 wk) for the climatic conditions in Israel, whereas the maximum average daily THI during the last 5 wk of gestation in the present study was 68.64.

Direct and maternal heritabilities for BiW by sPC1 are presented in Figure 1. The minimum and maximum sPC1 represent cold stress and HS, respectively, for the dams in late gestation. The midpoint for sPC1 (~0) reflects the thermoneutral zone. The direct heritabilities were quite constant, close to 0.33 in the course of sPC1, and the corresponding standard errors were 0.01. In statistical pedigree-based models and ignoring HS effects, the direct heritabilities for BiW were 0.46 (Yin and König, 2018) and 0.47 (Yin and König, 2019). Accordingly, the maternal heritabilities were larger in the 2 previous studies (0.14 and 0.19) compared with the estimates in the current study with 0.07 to 0.08 across sPC1. Different direct and maternal heritabilities across studies might be explained due to the following reasons. First, we only considered dams with repeated calf measurements, enabling an accurate modeling of the maternal permanent environmental contribution. Second, we observed an effect in modeling herd contemporaries. In model 2, we considered the fixed HY effect plus a random HYM effect, allowing a clear separation of environmental and genetic trend effects (Yin et al., 2012). Model superiority was indicated via increased 2log-likelihood values for our chosen model 2 with more precise contemporary group effects. Third, additional consideration of THI during late gestation contributes to a more detailed characterization of the environmental component, also capturing parts of the trait variance in the offspring generation.

**Figure 1:**
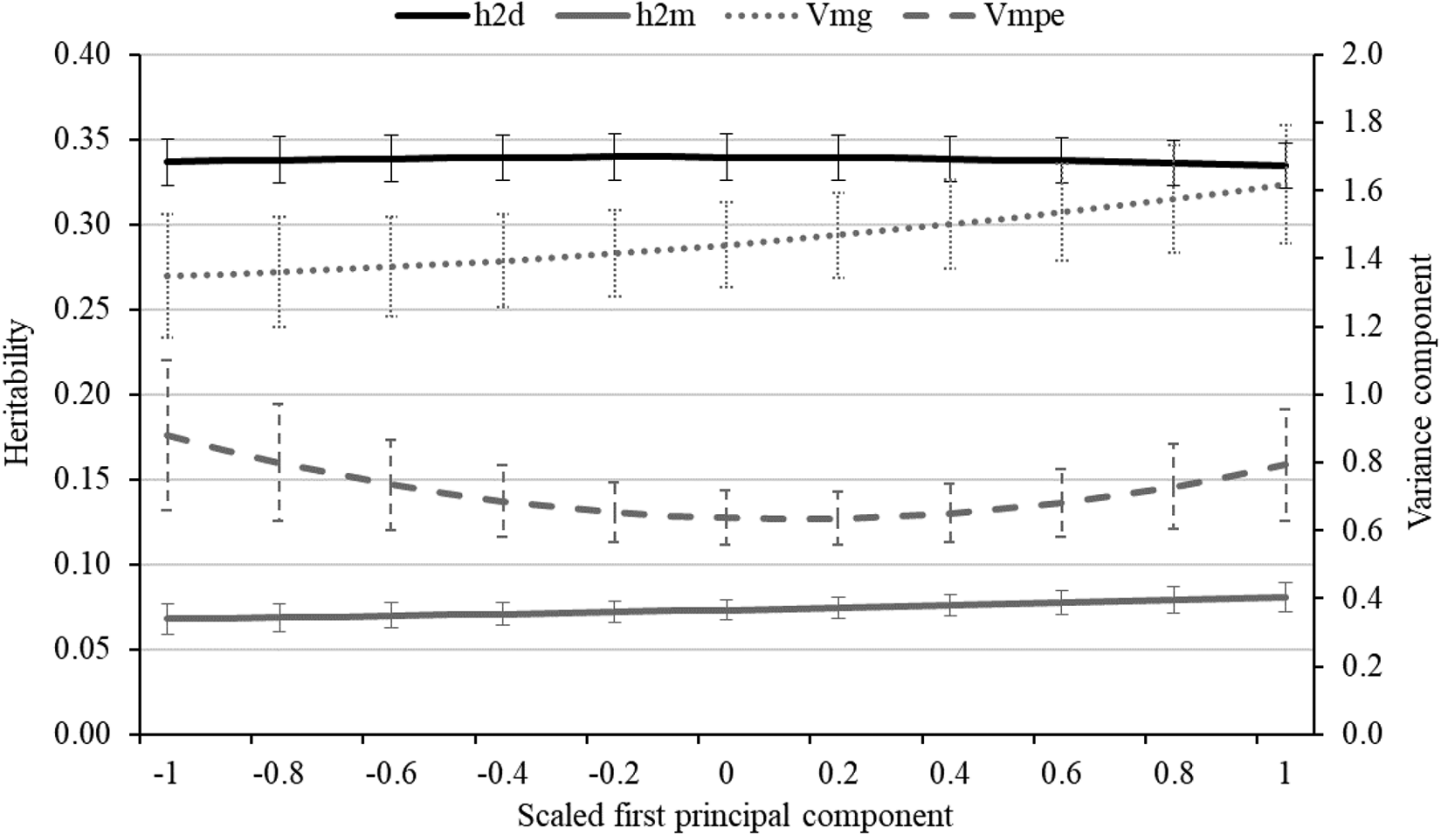
Direct (h2d) and maternal heritabilities (h2m) for calf birth weight across the scaled first principal component considering daily temperature and humidity index during the last 0 to 56 d of gestation. The corresponding SE are indicated as stripes. Vmg = variance for the maternal genetic effect; Vmpe = variance for the maternal permanent environmental effect.

The maternal heritabilities slightly increased from 0.07 at minimum sPC1 to 0.08 at maximum sPC1 (Figure 1), but the standard error of 0.01 implies that the increase was not significant. The direct and maternal heritabilities by sTHI for the single weeks were quite similar to the heritabilities reported for sPC1. The maternal genetic variance for BiW slightly increased from 1.35 to 1.62 along sPC1 (Figure 1). With regard to the maternal permanent environmental variance, the estimates were lowest in the thermoneutral zone, and largest at both extreme ends of the climatic gradient. However, the differences for maternal genetic and maternal permanent environmental variances along sPC1 were not significant.

The genetic correlations between maternal effects for BiW at the maximum sPC1 with BiW at the remaining sPC1 were larger than 0.95 (Figure 2), disproving possible interactions between maternal genetic effects and prenatal HS during late gestation. On the contrary, Yin et al. (2022) proved maternal genotype by time-lagged climate interactions for calf diseases. Hence, the responses of maternal genetic effects to changes in prenatal THI seem to be trait specific, with stronger interactions for low heritability disease traits. The maternal environmental correlations (**r_mpe_**) substantially declined with increasing distances between maximum sPC1 and the remaining sPC1. The r_mpe_ was minimal (0.56) for BiW at the maximum and minimum sPC1, indicating pronounced interactions between maternal permanent environmental effects and the HS status of the dams during the dry period. The correlations were generally lower than 0.8 between BiW at the maximum sPC1 with BiW at all sPC1 <−0.2. Such results indicate that dams suffering or not suffering HS during the dry period provide inconsistent in utero environments to their fetuses. Therefore, the phenotypic BiW decline in offspring with increasing prenatal THI is more likely due to the maternal permanent environmental rather than maternal genetic effects. The proportions of BiW variances explained by the maternal permanent environmental component were quite small, ranging from 3.22% in the thermoneutral zone (sPC1 = 0.1) to 4.43% under cold stress (sPC1 = −1). Hence, the implementation of cooling techniques during the dry period will slightly contribute to a stronger fetus body weight development. However, from a practical perspective, these BiW differences are quite small, and a strong BiW increase might induce calving difficulties. The possible calving difficulties versus disease resistance trade-offs, suggest an intermediate BiW. The THI in the present study calculated based on climate data from public weather stations might differ from the climate conditions in the cow barn because of the farm-specific management and husbandry conditions. Nevertheless, in the case of large data sets, Freitas et al. (2006) indicated unbiased genetic evaluations for heat tolerance when using meteorological data collected from the nearest public stations.

**Figure 2:**
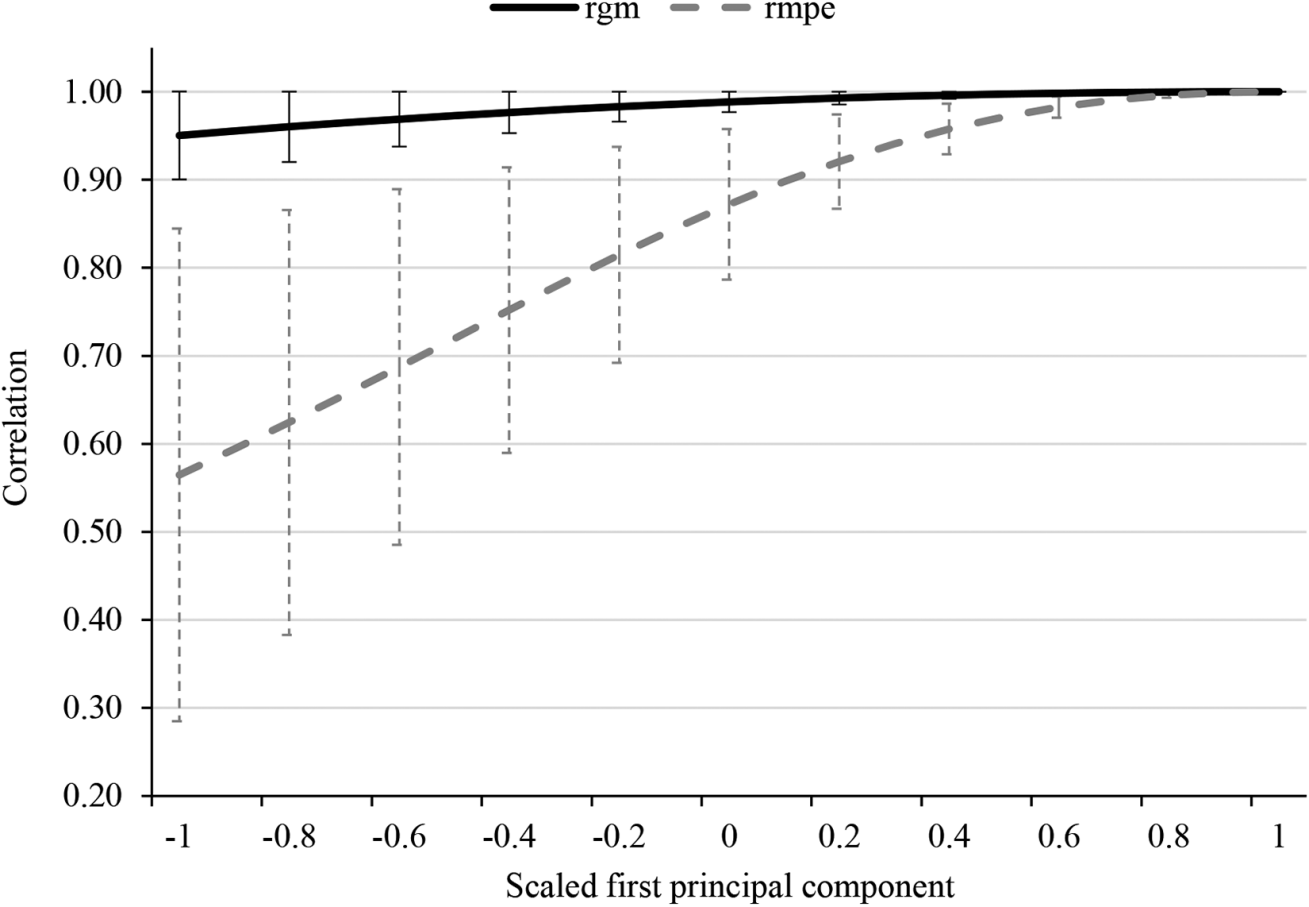
Correlations between birth weight at maximum scaled first principal component (sPC1; with heat-stressed dams) and remaining sPC1 (without heat-stressed dams) for maternal genetic (rmg) and maternal permanent environmental effects (rmpe). The corresponding SE are indicated as stripes.

In conclusion, prenatal HS, in particular high THI during WK8 of gestation, has detrimental effects (as indicated via negative regression coefficients) on BiW in offspring. Direct and maternal heritabilities for BiW were quite similar in HS and non-HS environments. The large correlations between the maternal genetic effects for BiW at the maximum HS level (sPC1 = 1) and the remaining levels indicate the absence of genotype by time-lagged climate interactions. The moderate correlations between maternal permanent environmental effects for BiW at sPC1 = 1 (maximum HS) with BiW at sPC1 <−0.2 explain the negative regression coefficients of BiW in response to prenatal THI. Therefore, the decline in BiW up to 0.63 kg per scaled prenatal THI is more likely caused by maternal permanent environmental effects instead of genetics. From the across-generation perspective, such result suggests the implementation of cooling technique instead of genetic evaluations for heat tolerance. From a practical farming perspective, maternal HS effects on offspring BiW were quite small.
